# Predictors of response to cardiac resynchronization therapy on pre-implantation cardiovascular magnetic resonance imaging

**DOI:** 10.1186/1532-429X-15-S1-E40

**Published:** 2013-01-30

**Authors:** Usha Manian, Raymond Yee, Immaculate Nevis, David McCarty, John Stirrat, David Scholl, Lorne J Gula, Peter Leong-Sit, Maria Drangova, James A White

**Affiliations:** 1 Medicine, London Health Sciences Centre, London, ON, Canada; 2 Imaging Research Laboratory, Robarts Research Institute, Western University, London, ON, Canada; 3 Medical Biophysics, Western University, London, ON, Canada

## Background

Cardiac resynchronization therapy (CRT) is an established treatment for severe heart failure. However, up to 40% of patients do not respond. While regional scar distribution has received focused attention, the predictive utility of global markers of remodeling and irreversible injury has not been well explored.

## Methods

Sixty-eight patients receiving CRT underwent pre-implant cardiovascular MRI followed by serial echocardiography at 3 and 6 months. Blinded measurement of Left Ventricular (LV) and Right Ventricular (RV) chamber dimensions, volumes and mass were performed from short axis cine datasets. LV dysynchrony was measured by septal to lateral wall delay. Total LV scar burden was determined from Late Gadolinium Enhancement (LGE) images using manual contour tracing of endocardial and epicardial borders with application of a signal threshold ≥5SD above reference myocardium. Response to CRT was defined as a reduction in LV end-systolic volume (ESV) ≥15% at 6 months.

## Results

The mean age was 66.3 ± 8.9 years with a mean LV Ejection fraction (EF) of 25.2 ± 7.2%. Overall, 47 patients (69%) responded. Among all baseline measures LVEDV (p=0.03), LVESV (p=0.045), RV EF (p=0.0349) and total scar burden (p=0.018) were the only significant predictors of CRT response. Multivariate analysis showed total scar burden to be the only independent predictor of CRT response (p=0.015).

## Conclusions

Pre-implantation MRI offers markers for the prediction of response to CRT. Of these, total scar burden appears to be an independent predictor of response and may be of assistance in the selection of optimal candidates.

## Funding

None

